# Early life factors, childhood cognition and postal questionnaire response rate in middle age: the Aberdeen *Children of the 1950s *study

**DOI:** 10.1186/1471-2288-5-16

**Published:** 2005-05-05

**Authors:** Yuji Nishiwaki, Heather Clark, Susan M Morton, David A Leon

**Affiliations:** 1 Department of Epidemiology and Population Health, London School of Hygiene & Tropical Medicine, London, UK; 2 Dugald Baird Centre, University of Aberdeen, Aberdeen, UK; 3 Liggins Institute and School of Population Health, University of Auckland, Auckland, New Zealand

## Abstract

**Background:**

Little is known about the relationship between early life factors and survey response in epidemiological studies of adults.

**Methods:**

The *Children of the 1950s *cohort is composed of 12,150 children (boys 51.7%) born in Aberdeen 1950–56 and in primary schools in the city in 1962. Information on birth weight, gestational age, growth, behaviour and socio-economic position at birth and in childhood were obtained from contemporaneous records. Cognitive test scores at ages 7,9 and 11 years were also available from school records. The outcome was response to a postal questionnaire sent (2001–2003) to surviving cohort members in middle age.

**Results:**

Of 11,282 potentially mailed subjects, 7,183 (63.7%) returned questionnaires. Response rates were highest among females, and those whose parents were married at birth, were in a non-manual social class at birth or in childhood, had fewer siblings, were taller and heavier in childhood for their age and had lower Rutter B behavioural scores. Childhood cognitive test scores at every age were strongly and positively related to the response rate to a postal questionnaire independently of other early life factors monotonically across the entire range of test scores. Those in the bottom fifth at age 11 had a response rate of 49% while those in the top fifth 75%.

**Conclusion:**

The strength and consistency of the association of childhood cognition with questionnaire response rate in middle age is surprisingly large. It suggests that childhood cognition across the entire normal range is a powerful influence on the complex set of later behaviours that comprise questionnaire response. The extent of possible response bias in epidemiological studies of the associations between childhood characteristics (particularly those related to cognition) and later health is probably larger than is generally realised, at least in situations where the survey instrument is a postal questionnaire.

## Background

The potential bias introduced by non-response or non-participation is an issue that affects much of observational epidemiology. Extending our understanding of the factors that influence non-response is thus an important area of research in its own right [1,2]. It is well known that many characteristics measured in adulthood such as gender, age, socio-economic position, education, health status and smoking habits are associated with response rate [2-10]. However, very little is known about the association of characteristics in infancy and childhood with response or recruitment rates in adulthood. This is partly because there are relatively few contexts in which unbiased data on early life characteristics are available for the total population irrespective of later response behaviour. The increasing interest in life-course epidemiology [11,12], in which adult disease is related to factors across an individual's life course, makes an investigation of these early-life influences on response rate particularly pertinent at this time.

In this paper we look at the influence of a range of infant and childhood characteristics on adult questionnaire response rates in a large cohort study. We pay particular attention to childhood cognition as we are also interested in the broader question of why adult health is related to childhood cognition [13-15]. A priori one might expect childhood cognition to be related to response rates, partly through its link with educational attainment, that is known to be strongly associated with response rates. However, to date there have been very few studies of the association of factors in early life, including cognition, and questionnaire or survey response in adult life. In an analysis of the UK 1946 national birth cohort, Wadsworth et al. reported that lower cognitive score at 8 years of age was associated with previous refusal or failure to contact by the study nurse at age 53 years [16]. However, the findings from this paper may not be readily generalizable, as the study is unusual in that it involved repeated contact with participants over their entire life time. More typically, life-course studies have information from infancy and childhood from historic sources, and then attempt a first survey follow-up well into adult life.

We have used the Aberdeen *Children of the 1950s *historical cohort study to investigate the association between early life factors, including childhood cognition, and response to a postal questionnaire conducted in middle age that represented the first attempt to make direct contact with the study subjects since childhood.

## Methods

### The Aberdeen Children of the 1950s study

The background to this historical cohort study has been previously described [17]. Briefly, this cohort is a large subset of the Aberdeen Child Development Study (ACDS) [18]. This was conducted in the early 1960s to estimate the population prevalence of mental subnormality in children and to investigate its etiology. Aberdeen was chosen as both obstetric and educational records were of a high standard and the population was relatively stable.

The ACDS was comprised of all 15 thousand children born 1950–56 who were in primary school in Aberdeen, Scotland in December 1962. In order for the ACDS to achieve its main objectives, particular care was taken by the original investigators to include all children of primary school age in Aberdeen regardless of their cognitive function. Thus children in schools for individuals with moderate or severe learning difficulties were included as well as those in ordinary primary schools. However, an unknown but small number of children with very severe learning difficulties who did not attend any school will not have been included in the original cohort.

In December 1962 these children (aged 7–12 years) were administered a range of age-appropriate reading tests and were requested to provide their own and parental demographic information. In March 1964 they were resurveyed and behavioral information was collected by class teachers using a detailed behavioural inventory for each child. Information was also obtained retrospectively from school test records (cognition at 7 and 9 years of age) and school medical records such as height and weight. Cognition scores at 11 years of age were obtained prospectively for most children who were aged 8 years or more in December 1962.

The *Children of the 1950s *cohort is comprised of the 12,150 individuals (males 51.7%) in the ACDS who were born in Aberdeen and had been successfully linked to information from obstetric records held in the Aberdeen Maternity and Neonatal Databank. The process of revitalising this cohort was begun in 1998. This included tracing of the vital status and area of residence and address of the study participants and attempting to mail a questionnaire to those subjects believed to be currently resident in the UK. This 21 page questionnaire covered a range of topics including living conditions, occupation, education, income, height and weight, health, health-related behaviours, and parental vital status. A copy of the questionnaire is available from the authors on request. This was the first time that a direct attempt had been made to contact any of the cohort members since childhood, with the exception of a small subgroup (less than 1 in 5) who had been followed up in various investigations of respiratory function since the mid-1980s, known collectively as the WHEASE study [19,20].

### Tracing of cohort members

The tracing of the current vital status and area of residence of the cohort members was carried out by the National Health Service Central Register (NHSCR) for Scotland, England and Wales. Using name, date of birth and the fact that the subjects were known to have been resident in Aberdeen in 1962, the NHSCR was able to identify all but 136 (1.1%) of subjects in their records.

### Questionnaire mailing

The mailing of questionnaires to cohort members was begun in May 2001. For confidentiality reasons, the primary mailing to subjects believed to be resident in Scotland (n = 9,261) was carried out on our behalf by the Information and Statistics Division (ISD) of the National Health Service in Scotland who had access to current addresses. Those not responding to this initial mailing were re-mailed by ISD in August 2001. For those resident in England and Wales (n = 1,062), local health authorities undertook the mailing exercise (February – August 2002). A small proportion of subjects (n = 328) were given the questionnaires as a part of the WHEASE study. Those not returning a questionnaire after a mailing from ISD or health authorities and those who were not mailed through these channels for other reasons (mainly that a precise address could not be determined by ISD) were sent to the Driver and Vehicle Licensing Agency (DVLA) (n = 4,355). They agreed to send out questionnaires to those people who they could identify in their database (October 2002). However, in order to protect confidentiality DVLA were not able to tell us which subjects they were successful in identifying and sending a questionnaire to. Finally, between December 2002 and February 2003 a small number of non-responders (n = 556) were sent questionnaires via their siblings who had already responded earlier on. However, we were unable to ascertain how many of these questionnaires were actually delivered to their intended recipients.

**Figure 1:**
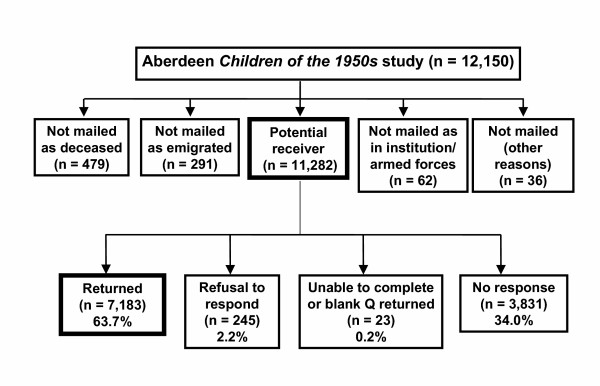
**Profile of mailing questionnaire in the Aberdeen *Children of the 1950s *study**. Potential receiver includes subjects who were not traced through National Health Service Central Registry or cancelled General Practice registration as well as surviving cohort members.

Figure 1 summarizes the final outcomes of the questionnaire survey responses. We attempted to contact all subjects regardless of their early life characteristics including cognition. However, of our original 12,150 subjects we did not mail those who were dead (n = 479), were known to have emigrated from the UK (n = 291) or were members of the armed forces (or family of those in the armed forces) or those who were in prison or in a long stay psychiatric hospital (n = 62). Of those we tried to mail through the national health service for a small number (n = 27) the Health Authority or Health Board or General Practitioner refused to forward the questionnaire to the subjects' address. Finally we did not attempt to mail the very small number subjects (n = 9) who were residents of the Western Isles of Scotland or North Ireland.

### Cognition and other factors in early life

The Moray House Picture Intelligence Test, Schonell and Adams Essential Intelligence Test and Moray House Test were used as cognitive tests at 7, 9 and 11 years respectively. The Moray House Test at 11 consisted of two ability tests (verbal reasoning 1 & 2) and two attainment tests (Arithmetic, English). The scores from these tests were combined together with a teacher's estimate to yield a "total transfer score"[21] that played a crucial role in determining the type of secondary school the child went onto. All these cognitive test scores were standardised to give means of 100 and standard deviations (SDs) of 15 using national norms for cognition at 7 and 9 and local norms for cognition at 11.

The following additional factors were also considered in this study. Sex and year of birth of children were obtained from the survey in 1962. Birth weight was measured in pounds and categorized into 4 groups (<6.5, 6.5–7.5, 7.5–8.5 and ≥8.5). Gestational age was categorized into 5 groups (≤38, 39, 40, 41 and ≥42 completed weeks of gestation). Height for age was derived from height and age at first medical examination, which was at around 5 years of age. A sex-specific z-score was calculated for each child within 3 month age bands. Using this z-score, subjects were classified into 4 groups (≤-1, -1 to 0, 0 to 1, ≥1 SD). Weight for age z-score categories were derived using the same approach. As a behavioural factor, Rutter B score was assessed by the children's class teachers based on 26 questions. A score of 9 or over was defined as indicating psychological disturbance [22,23]. In this analysis, scores were divided into four (0, 1–2, 3–8, and ≥9). Father's occupational class at birth and in 1962, number of siblings in 1962 and mother's marital status at birth were also obtained.

### Ethical approval

The revitalisation of the *Children of the 1950s *cohort including the questionnaire survey was approved by the Scottish Multi-Centre Research Ethics Committee, the Scottish Privacy Advisory Committee and various local ethics committees in Scotland and England and Wales.

### Statistical analysis

Absolute response rates (or more properly response proportions) were examined in cross-tabulations according to explanatory factors of interest. The associations between response proportions and explanatory factor were quantified in terms of odds ratios. Subjects who returned a questionnaire were considered as responders even if the questionnaire included some degree of item non-response. However, subjects who returned a questionnaire that was completely blank were classified as non-responders. In the original data, children who were considered not capable of being meaningfully tested using the standard instruments because of their degree of cognitive impairment (n = 8) were arbitrarily allocated a score of 50. As exclusion of these subjects did not change results, we included them in the analyses.

Logistic regression was used to model the association of explanatory variables with response coded as a binary variable. The trend in odds ratios were assessed by the score test. As cognitive test scores for females were slightly higher than those for males, sex-specific quintiles of cognitive test scores were used for categorization. Sex-adjusted odds ratios for a one SD increase in cognition scores at 7, 9 and 11 years were compared with those additionally adjusted for other early life factors. In addition, sex-adjusted odds ratios for cognition scores were compared with those adjusted for cognition scores at different ages. Interactions among early life factors were tested using the likelihood ratio test. All analyses were conducted using STATA 8 [24].

## Results

The average age (range) of the subjects at the start of the questionnaire survey in May 2001 was 48.1 years (45.1 to 51.3). Of 11,282 (5,742 males, 5,540 females) potentially mailed subjects, 7,183 (3,432 males, 3,751 females) returned questionnaires. This constituted an overall response rate of 63.7%. A total of 3,831 (34.0%) did not respond, 245 (2.2%) refused to participate and 23 (0.2%) were unable to complete or returned blank questionnaires (Figure 1). Females had an appreciably higher response rate (67.7%) than males (59.8%). Table 1 summarises the association of response rate with key explanatory variables for males and females separately. In males and females response rates were higher for subjects whose mothers were married and whose fathers were in a non-manual social class when they were born. Response rates were also higher among those who were had fewer siblings, were taller and heavier for their age in childhood and did not exhibit symptoms of behavioural disorders (indicated by a low Rutter B score). Tests for interaction of each explanatory variable with sex were all non-significant with the exception of mother's marital status at birth where the married category was associated with a greater response rate in females compared to males. There was no association of response rate with year of birth or birth weight or gestational age (not shown).

**Table 1:** Questionnaire response rates by early life factors

	Male				Female			
	Number	Response rate (%)	Odds ratio	(95% CI)	Number	Response rate (%)	Odds ratio	(95% CI)
**Sex**								
Male	5,742	59.8	1.00					
Female	5,540	67.7	1.41	(1.31 to 1.52)				
**Birth weight (lbs)**								
<6.5	1,118	58.7	1.00		1,451	65.0	1.00*	
6.5–7.5	1,874	588	1.00	(0.86 to 1.17)	2,061	68.3	1.16	(1.01 to 1.34)
7.5–8.5	1,809	60.7	1.09	(0.93 to 1.27)	1,480	69.6	1.23	(1.06 to 1.44)
≥8.5	929	61.0	1.10	(0.92 to 1.32)	539	68.3	1.16	(0.94 to 1.43)
Missing†	12	83.3			9	22.2		
**Mother's marital status at birth**‡								
Single/widowed/divorced	175	52.0	1.00		190	50.5	1.00	
Married	5,564	60.0	1.39	(1.02 to 1.87)	5,349	68.3	2.11	(1.58 to 2.83)
Missing†	3	66.7			1	0.0		
**Father's social class at birth**								
Unemployed/disabled/deceased	297	52.9	1.00*		336	56.9	1.00*	
IV/V	1,765	55.9	1.13	(0.88 to 1.45)	1,633	63.4	1.32	(1.04 to 1.67)
III Manual	2,486	59.7	1.32	(1.04 to 1.68)	2,442	68.4	1.64	(1.30 to 2.07)
III Non-manual	633	65.9	1.72	(1.30 to 2.29)	613	73.9	2.15	(1.62 to 2.86)
I/II	561	69.2	2.00	(1.49 to 2.68)	515	77.9	2.67	(1.96 to 3.63)
Missing†	0				1	0.0		
**Father's social class in 1962**								
Unemployed/disabled/deceased	341	46.6	1.00*		357	53.5	1.00*	
IV/V	1,424	55.4	1.42	(1.12 to 1.80)	1,419	64.3	1.57	(1.24 to 1.98)
III Manual	2,419	60.0	1.72	(1.36 to 2.16)	2,291	68.0	1.84	(1.47 to 2.31)
III Non-manual	701	67.3	2.36	(1.80 to 3.09)	630	73.2	2.37	(1.79 to 3.13)
I/II	776	67.8	2.41	(1.85 to 3.14)	781	76.6	2.84	(2.16 to 3.73)
Missing	81	43.2	0.87	(0.54 to 1.42)	62	50.0	0.87	(0.51 to 1.48)
**Number of siblings in 1962**								
5 or more	1,041	50.4	1.00*		946	59.2	1.00*	
4	938	57.7	1.34	(1.12 to 1.60)	942	65.2	1.29	(1.07 to 1.56)
3	1,424	61.5	1.57	(1.34 to 1.85)	1,420	70.9	1.67	(1.41 to 1.99)
2	1,749	64.2	1.76	(1.50 to 2.06)	1,677	71.3	1.71	(1.45 to 2.03)
1	545	64.6	1.79	(1.45 to 2.22)	510	68.8	1.52	(1.21 to 1.91)
Missing	45	35.6	0.54	(0.29 to 1.00)	45	53.3	0.79	(0.44 to 1.42)
**Height for age at 1st medical exam 1962**								
≤-1	734	54.4	1.00*		872	62.3	1.00*	
-1 to 0	1,882	57.1	1.12	(0.94 to 1.33)	1,969	66.0	1.17	(1.00 to 1.39)
0 to 1	2,139	62.3	1.39	(1.17 to 1.65)	1,775	69.9	1.40	(1.18 to 1.67)
≥1	737	66.9	1.70	(1.37 to 2.10)	723	73.7	1.70	(1.37 to 2.11)
Missing	250	52.8	0.94	(0.70 to 1.25)	201	67.7	1.27	(0.92 to 1.75)
**Weight for age at 1st medical exam (SD)**								
≤-1	870	56.7	1.00*		795	63.9	1.00*	
-1 to 0	2,045	59.5	1.12	(0.96 to 1.32)	2,109	65.5	1.07	(0.91 to 1.27)
0 to 1	1,810	59.2	1.11	(0.94 to 1.31)	1,667	70.0	1.32	(1.10 to 1.58)
≥1	767	67.8	1.61	(1.31 to 1.97)	769	72.7	1.50	(1.21 to 1.87)
Missing	250	52.8	0.86	(0.65 to 1.13)	200	67.5	1.17	(0.84 to 1.63)
**Rutter B behavioral score**								
9+	595	47.4	1.00*		320	57.2	1.00*	
3–8	1,736	56.2	1.42	(1.18 to 1.72)	1,448	61.5	1.19	(0.93 to 1.53)
1–2	1,517	63.8	1.96	(1.61 to 2.38)	1,637	69.6	1.72	(1.34 to 2.20)
0	1,720	65.7	2.13	(1.75 to 2.58)	1,990	73.3	2.06	(1.61 to 2.63)
Missing	174	44.3	0.88	(0.63 to 1.24)	145	54.5	0.90	(0.60 to 1.33)

* p < 0.05 for the score test for trend (missings were not included).

† We did not calculate odds ratio and 95% confidence interval due to small sample size.

‡ p < 0.05 for the interaction with sex tested by using likelihood ratio test in logistic regression.

**Figure 2:**
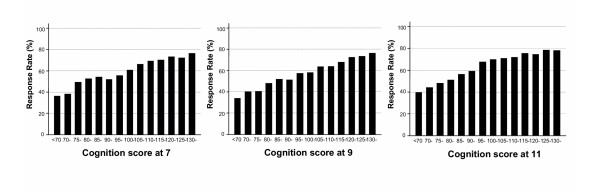
**Response rates by cognition test scores at 7, 9 and 11 for males and females combined**. Five point bands were used for cognition scores.

The associations of response rate with cognitive score at ages 7, 9 and 11 years for both sexes combined are shown in Figure 2. The almost monotonic increase in questionnaire response rate with each 5 point increase in score at each age is very striking. Response rates by quintile of cognitive score are summarised separately by sex in Table 2. Response rates increased progressively at all ages from the bottom to the top fifth of cognitive test score in both sexes. Each of the four components of cognition measured at 11 years (verbal reasoning × 2, English and arithmetic) showed similar associations with response and were adequately summarised by association with the total score. There was no evidence of a statistically significant interaction between sex and cognitive scores at any age, and thus in the remaining analyses we present results for both sexes combined.

**Table 2:** Questionnaire response rates by sex-specific cognition score quintiles

	Male (n = 5,742)						Female (n = 5,540)					
		
	Test score						Test score					
										
	Mean	Range	Number	Response rate (%)	Odds ratio	(95% CI)	Mean	Range	Number	Response rate (%)	Odds ratio	(95% CI)
**Cognition at 7**												
Lowest quintile	84	50, 93	1,111	48.2	1.00*		84	50, 93	1,006	52.9	1.00*	
2nd lowest	98	94, 102	1,046	54.0	1.26	(1.07 to 1.50)	98	94, 103	1,058	60.3	1.35	(1.14 to 1.61)
Middle	106	103, 110	1,128	59.3	1.57	(1.33 to 1.86)	107	104, 111	1,023	72.7	2.38	(1.97 to 2.87)
2nd highest	115	111, 120	1,126	67.4	2.23	(1.87 to 2.65)	116	112, 121	1,099	73.6	2.49	(2.06 to 3.00)
Highest quintile	129	121, 153	1,173	70.3	2.55	(2.14 to 3.05)	130	122, 166	1,080	78.4	3.24	(2.66 to 3.95)
Missing			158	50.0	1.08	(0.77 to 1.50)			274	66.1	1.73	(1.31 to 2.29)
**Cognition at 9**												
Lowest quintile	85	50, 95	1,047	45.7	1.00*		89	60, 98	1,002	54.3	1.00*	
2nd lowest	102	96, 106	998	57.2	1.59	(1.34 to 1.90)	104	99, 107	921	62.4	1.40	(1.17 to 1.68)
Middle	111	107, 115	1,207	60.5	1.82	(1.54 to 2.16)	111	108, 115	1,078	68.9	1.87	(1.56 to 2.24)
2nd highest	119	116, 124	1,049	64.9	2.20	(1.84 to 2.63)	119	116, 123	1,047	74.5	2.46	(2.03 to 2.98)
Highest quintile	135	125, 174	1,139	72.2	3.09	(2.57 to 3.71)	133	124, 176	1,110	79.1	3.19	(2.61 to 3.88)
Missing			302	49.7	1.17	(0.91 to 1.52)			382	60.5	1.29	(1.01 to 1.64)
**Cognition at 11**												
Lowest quintile	78	54, 86	920	45.5	1.00*		80	47, 87	843	52.4	1.00*	
2nd lowest	91	86, 95	903	54.6	1.44	(1.19 to 1.73)	92	88, 97	890	63.3	1.56	(1.29 to 1.89)
Middle	99	96, 103	934	63.6	2.09	(1.73 to 2.52)	101	97, 104	845	74.1	2.59	(2.10 to 3.20)
2nd highest	108	104, 113	931	67.2	2.45	(2.02 to 2.98)	109	105, 114	881	74.6	2.66	(2.16 to 3.28)
Highest quintile	121	113, 144	922	71.4	2.98	(2.44 to 3.64)	121	114, 146	874	78.6	3.33	(2.68 to 4.15)
Missing			1,132	56.7	1.57	(1.32 to 1.87)			1,207	64.3	1.63	(1.37 to 1.95)

*p < 0.05 for the score test for trend (missings were not included).

Sex-adjusted odds ratios for one SD increase in cognition scores at 7, 9 and 11 were slightly attenuated (less than 6%) by adjustment for socio-economic position, height and weight for age and Rutter B score (Table 3) but nevertheless remained substantial. Cognitive test scores at ages 7, 9 and 11 years are highly correlated (Pearson correlation coefficients: 0.74 to 0.88). How far the associations of response rate with cognitive score at each age are independent is also examined in Table 3. Nearly all of the effects at ages 7 and 9 were removed by adjustment for cognitive test score at age 11 years. In contrast, the effect of cognition at 11 was only slightly attenuated by adjustment for cognitive test score at 7 or 9.

**Table 3:** Odds ratios for questionnaire response by cognition scores with adjustment for other early life factors and cognition at different ages

	**Cognition at 7**		**Cognition at 9**		**Cognition at 11**	
	OR†	(95% CI)	OR†	(95% CI)	OR†	(95% CI)
Adjusted for						
Sex	1.42	(1.36 to 1.49)	1.50	(1.43 to 1.57)	1.55	(1.47 to 1.62)
Sex, Marital status at birth	1.42	(1.35 to 1.48)	1.49	(1.42 to 1.56)	1.54	(1.47 to 1.62)
Sex, Father's occupation at birth & in 1962, Number of children	1.34	(1.27 to 1.41)	1.42	(1.35 to 1.49)	1.48	(1.40 to 1.56)
Sex, Height & Weight at 1st medical exam	1.40	(1.34 to 1.47)	1.48	(1.41 to 1.55)	1.53	(1.46 to 1.61)
Sex, Rutter B behavioral score	1.36	(1.29 to 1.42)	1.43	(1.36 to 1.51)	1.50	(1.42 to 1.58)
Sex, All early life factors‡	1.29	(1.22 to 1.36)	1.37	(1.30 to 1.45)	1.43	(1.36 to 1.52)
						
Sex, Cognition at 7		-	1.36	(1.27 to 1.46)	1.46	(1.37 to 1.57)
Sex, Cognition at 9	1.14	(1.06 to 1.22)		-	1.44	(1.30 to 1.59)
Sex, Cognition at 11	1.08	(1.01 to 1.15)	1.09	(0.98 to 1.20)		-

* Due to missing data for some variables, this analysis was restricted to a subset of 8,452 subjects.

† Odds ratio for one standardized deviation increase in each cognition score using logistic regression.

‡ Adjusted for all early life variables (marital status at birth, father's occupation at birth & in 1962, number of children, height & weight at 1st medical exam, and Rutter B behavioural score)

## Discussion

We have found that a wide range of characteristics in infancy and childhood were associated with questionnaire response rate. Cognitive test score in childhood was particularly strongly related to the probability of responding to the questionnaire survey independently of other factors, with an almost monotonic increase in response rate, with no evidence of a threshold effect. At age 11 response rates among those in the top fifth of the cognitive test score distribution had a response rate that in absolute terms was 25% higher than in the bottom fifth.

Although the possibility of obtaining biased samples in postal questionnaires with incomplete response rates has been well documented in the survey and epidemiological literature [3,5,7,25,26], the emphasis has been mainly upon the determinants of response related to characteristics of subjects at the time they receive the survey instrument. Ours is the first study that has looked at early life influences on response rate to a postal questionnaire in middle age in a cohort not previously contacted in adult life. A similar analysis by Wadsworth and colleagues [16] of childhood influences on later continued participation in the British 1946 birth cohort, in which subjects had been repeatedly contacted throughout life, found that continued participation was least likely among those who had been in the most disadvantaged socio-economic circumstances in childhood and those with lowest cognitive test scores at 8 years of age. The sex-adjusted odds ratio for avoidable loss to follow-up at age 53 years in the top compared to the bottom quarter of childhood cognition was 0.52 (95% Confidence Interval; 0.42 to 0.64). However, these analyses did not involve any multi-variable adjustments, so it is unclear how far this association may be explained by confounding with socio-economic and other factors.

Finding an association between childhood cognition and questionnaire response rate in adult life was expected. However, the strength and consistency of this association, with almost monotonic increases across the entire range of test scores, are surprising. At least two explanations for this powerful effect should be considered. The first one would hypothesise a pathway via education. High cognitive test scores in childhood are highly predictive of higher levels of education. Subjects with higher level of education may have higher response rate because they may be more health conscious, more interested in research and not feel intimidated by a relatively substantial questionnaire. The second possibility is a more direct pathway, where cognitive ability such as a problem solving (akin to completing a 21 page questionnaire) played an important role. Our study was not able to definitively distinguish between these two pathways. However, it should be noted that the effect of cognition at 11 was independent of cognition at 7 and 9 years, whereas the effect of cognition at 7 was attenuated substantially by adjusting for cognition at 11. The test at age 11 explicitly included components that measured educational attainment (mathematics and English), and the overall score was used as one of the key criteria for determining secondary school. Thus cognitive score at 11 is more likely to reflect educational progress to that age than the score at 7 years, suggesting that our data are most consistent with the link between childhood cognition and response rate being via attained educational level.

Some limitations of this study should be noted. Firstly, bias by undelivered mail might be possible. We do not know which subjects actually received a questionnaire. If cognitive test score was positively related to the probability of actually receiving the questionnaire this would generate, or at least contribute to, the observed gradient. However, this seems unlikely. As we have shown elsewhere [17], the lower a person's cognitive test score the less likely it was that they moved away from the Aberdeen area, and thus the lower the probability that the NHS had an incorrect or out-of-date address for them. Secondly, our data set included an appreciable proportion of subjects who did not have a cognitive test score at 11 (n = 2,339, 20.7% of 11,282). However, 80% (n = 1,873) of those without score at 11 were missing it simply because they had not reached this age during the survey period. We repeated the multivariate analyses after excluding cognition at 11 and confirmed that odds ratios for cognition at 7 and 9 were essentially unchanged. Therefore, we do not believe that selection bias due to this missing data could explain the observed associations. Finally, it should be noted that our findings may not automatically apply to other survey methods such as telephone and face to face interview.

Any postal questionnaire survey should be designed and undertaken to achieve the maximum possible response rate. However, in many contexts there will remain major concerns about non-response bias. In these situations the powerful links that exist between early life characteristics and response needs to be taken into account when interpreting the data. This can be done in part by employing sensitivity analyses. Moreover, in some situations multiple imputation methods may also be useful[27,28].

## Conclusion

Our results indicate that the interpretation of associations between childhood and later life factors in life-course studies using postal questionnaires need to take account of the fact that factors in childhood can be strongly related to response rate. Particular caution is needed if the outcomes are related to childhood cognitive function. Sensitivity analyses that explore the extent of such biases are strongly recommended.

Quite apart from these methodological conclusions, the study finding shows that this simple and routinely collected information on cognition is extraordinarily predictive of the complex collection of later-life behaviours and circumstances that jointly generate the likelihood of a completed questionnaire being returned.

## Competing interests

The author(s) declare that they have no competing interests.

## Authors' contributions

DAL, SMM and HC designed the questionnaire survey. HC supervised the data collection. YN analyzed, interpreted the data and drafted the paper in consultation with the other authors. All authors read and approved the final manuscript.

## Pre-publication history

The pre-publication history for this paper can be accessed here:


